# 2205 Thrombotic complications in single ventricle reconstructions for single ventricle physiology congenital heart disease

**DOI:** 10.1017/cts.2018.307

**Published:** 2018-11-21

**Authors:** Michael H. White, Kavita Patel, Lazaros Kochilas, Robert F. Sidonio

**Affiliations:** 1 Emory University; 2 Leader Thrombosis Clinical and Research Programs, Aflac Cancer and Blood Disorders Center, Children’s Healthcare of Atlanta, Emory University; 3 Sibley Heart Center Cardiology, Emory University; 4 Aflac Cancer and Blood Disorders Center, Children’s Healthcare of Atlanta, Emory University

## Abstract

OBJECTIVES/SPECIFIC AIMS: Infants with single ventricle congenital heart disease (CHD) who undergo staged surgical reconstruction are among the pediatric patients at highest risk for thrombotic complications. Despite improvements in survival due to medical and surgical advancements, thrombotic complications are common and lead to increased morbidity and mortality, especially during the first two stages of surgical reconstruction. The burden of disease caused by thrombosis is not fully known, and the risk factors associated with thrombosis are not clear. Due to this knowledge gap, prevention of thrombosis with medication, a strategy called thromboprophylaxis, has not been standardized, leading to inadequate prevention of thrombosis. In order to understand the burden of thrombosis and then provide targeted thromboprophylaxis for thrombosis prevention, better characterization of thrombotic complications and the associated factors is needed. Hypothesis: I hypothesize that in infants with single ventricle CHD, the incidence of thrombosis will be more frequent after stage I Versus stage II reconstruction, despite the type of shunt used. Specific demographic, clinical, and surgical variables will be associated with an increased risk for thrombotic complications, and a model to predict which subset of infants is at increased risk will be developed. Specific Aim 1: Characterize the incidence of thrombotic complications at different time points from stage I through stage II of the single ventricle reconstruction (SVR) pathway and determine the demographic, clinical, and surgical factors associated with thrombosis in infants with single ventricle CHD. (1) Determine the incidence of thrombosis in infants with single ventricle CHD. (2) Compare the rate of thrombotic complications between the 2 most commonly used approaches for stage I reconstruction for the group of patients with hypoplastic left heart type of anatomy [modified Blalock-Taussig shunt (MBTS) vs. right ventricle to pulmonary artery shunt (RVPAS)]. (3) Determine the factors (demographic, clinical, and surgical) associated with thrombosis in infants with single ventricle CHD. Specific Aim 2: Determine which subset of infants with single ventricle CHD is at increased risk of developing thrombotic complications across the first 2 stages of surgical reconstruction. (1) Test the identified demographic, clinical, and surgical variables including, but not limited to, gestational age, sex, CHD diagnosis, baseline oxygen saturation, stage of reconstruction, shunt type, and other clinical data available in a univariable and multivariable analysis and study their potential interactions to construct a novel risk predictive model specific for single ventricle CHD. METHODS/STUDY POPULATION: To address the specific aims, I will utilize data from the SVR clinical trial public use data set. This data set includes a prospective cohort of infants, 0–14 months of age, enrolled from any of the 15 participating clinical centers from the years 2005 to 2009. Inclusion criteria for enrollment were diagnosis of hypoplastic left heart syndrome or related single, morphologic right systemic ventricle anomaly, planned Norwood procedure, and informed consent of parent or legal guardian. No additional subjects outside of this data set will be included. Exclusion criteria were a diagnosis of single, morphologic left ventricle anomaly, preoperative identification of anatomy rendering the MBTS or RVPAS technically impossible, and any other major abnormality or acquired extra-cardiac disorder that could independently affect the likelihood of the subject meeting the primary endpoint. The complication of stroke will be excluded from the analyses of factors associated with thrombosis. The complication of thrombosis as defined in this dataset is a composite of events that include arterial or venous thrombosis, thromboembolism, and pulmonary embolism. The data was collected in such a way that it will not be possible to separate these sub-types of thrombosis. Additional thrombotic events of interest are superior vena cava occlusion and inferior vena cava occlusion. Specific Aim 1: Patient data will be extracted from the SVR clinical trial public use dataset to characterize the incidence of thrombotic complications at different time points from stage I through stage II of the SVR pathway and determine the demographic, clinical, and surgical factors associated with thrombosis in infants with single ventricle CHD. In addition, I will compare the rates of thrombotic complications between the 2 most commonly used approaches for stage I palliation for the group of patients with hypoplastic left heart type of anatomy (MBTS vs. RVPAS) and will test the hypothesis that the risk of thrombotic complications is associated with the stage of palliative surgery (stage I vs. stage II). Specific Aim 2: We will test identified demographic, clinical, surgical, and newly identified variables in a univariable and multivariable analysis and study their potential interactions to construct a novel risk predictive model specific for single ventricle CHD. RESULTS/ANTICIPATED RESULTS: To determine feasibility for adequate numbers to be able to address the research aims, a preliminary analysis dataset was performed using a dataset from the Pediatric Heart Network. The PHN is a collaborative group of hospitals that participates in clinical research studies in children with CHD. For the SVR clinical trial, the PHN conducted a randomized clinical trial at 15 centers in North America between 2005 and 2009, prospectively enrolling infants with HLHS or single right ventricle anomalies who were to undergo the Stage I Norwood procedure. A total of 920 newborns were screened; 664 were medically eligible and 549 patients were randomized. The primary aim of the trial was to compare survival of infants randomized to receive either the Norwood procedure with the MBTS or the RVPAS. These patients were followed at specific time points, including from baseline (pre-Norwood), at the time of the Norwood procedure, between stage I and II, following stage II reconstruction, and at 14 months of age. At these time points, data were collected that includes demographic, radiologic, clinical, and surgical outcomes. Included in the clinical outcomes are complications, such a thrombosis. There was no screening process to assess for asymptomatic thromboses, suggesting that most, if not all, discovered thromboses were due to clinically relevant effects. A newer iteration of this study (SVRIII) expands the monitoring of this cohort until the Fontan stage at 2–6 years of age, but these data have not yet been released in the public use data set. A descriptive analysis of the frequency of thrombotic complications was assessed at each time point, as well as in aggregate. Data were extracted from the specific time periods of interest, identified as Pre-Norwood, during Norwood Hospitalization, in-between visits, and during Stage II Hospitalization. There were 549 infants who were randomized with available data to analyze. During the Norwood hospitalization, 37 infants had a thrombotic complication. Between Stage I and Stage II outpatient visits, 8 infants had a thrombotic complication. During Stage II hospitalization, 16 infants had a thrombus. Overall, 61 individual patients (11%) had a thrombotic complication. DISCUSSION/SIGNIFICANCE OF IMPACT: This study utilizing data from the Pediatric Heart will be the largest cohort ever utilized for characterizing thrombotic complications and determining the factors associated with thrombosis across the first and second stages of surgical reconstruction. More than 500 (n=549) subject’s data will be analyzed through the first two stages of reconstruction, while the largest analysis before this proposed analysis only included a total of 195 children. Notably, these prior studies did not include a comparison between the 2 shunt types in stage I reconstruction, leaving a gap in knowledge regarding the incidence of thrombosis comparing these groups. The analysis will be the first to address this gap and update the current literature. Preliminary data show that the overall incidence of thrombosis across the first 2 stages of surgical reconstruction was 11%, which is lower than the previously reported overall rates of 40%–50%. Despite the continued lack of evidence-based guidelines for thromboprophylaxis methods, the decreased overall rate is most likely due to more widespread practice of anticoagulation in general. Determining the factors associated with thrombosis across the first and second stages of surgical reconstruction will help identify those at risk. An innovative aspect of this analysis will be the use of disease-specific factors to develop a model to predict thrombosis. Unique factors include cardiac variables like ejection fraction, baseline oxygen saturation, shunt type (MBTS vs. RVPAS), and other echocardiographic parameters. While the use of thromboprophylaxis has been associated with decreased risk of thrombosis, there is no general consensus to guide thromboprophylaxis in this population, which can be burdensome and costly. Determining which subset of infants with single ventricle CHD are at increased risk of developing thrombotic complications will allow for the development of a prediction model to predict those at highest risk of developing a thrombotic complication. Developing a predictive model will be a novel way to identify patients at risk for thrombosis and will set the stage for targeted prevention of thrombosis.

